# The pervasive effects of timing of parental mental health disorders on adolescent deliberate self-harm risk

**DOI:** 10.1371/journal.pone.0220704

**Published:** 2019-08-14

**Authors:** Nan Hu, Catherine L. Taylor, Rebecca A. Glauert, Jianghong Li

**Affiliations:** 1 Telethon Kids Institute, The University of Western Australia, Nedlands, Western Australia, Australia; 2 School of Population and Global Health, The University of Western Australia, Nedlands, Western Australia, Australia; 3 Centre for Child Health Research, The University of Western Australia, Nedlands, Western Australia, Australia; 4 WZB Berlin Social Science Centre, Berlin, Germany; International Telematic University Uninettuno, ITALY

## Abstract

Children whose parents have mental health disorders are at increased risk for deliberate self-harm (DSH). However, the effect of timing of parental mental health disorders on adolescent DSH risk remains under-researched. The aim of this study was to investigate how parental hospital admissions for mental health disorders and/or DSH in different developmental periods impact on the child’s DSH risk in adolescence. A nested case-control sample was compiled from a total population cohort sample drawn from administrative health records in Western Australia. The sample comprised 7,151 adolescents who had a DSH-related hospital admission (cases), and 143,020 matched controls who hadn’t had a DSH-related hospital admission. The occurrence of parental hospital admissions related to mental health disorders and/or DSH behaviours was then analysed for the cases and controls. The timing of the parental hospital admissions was partitioned into four stages in the child’s life course: (1) pre-pregnancy, (2) pregnancy and infancy, (3) childhood, and (4) adolescence. We found that adolescents of a parent with mental health and/or DSH-related hospital admissions in all developmental periods except pregnancy and infancy were significantly more likely than controls to have a DSH-related hospital admission. Compared to parental hospital admissions that occurred during childhood and adolescence, those that occurred before pregnancy conferred a higher risk for adolescent DSH: adjusted odds ratio (aOR) = 1.25 for having only one parent hospitalised and 1.66 for having both parents hospitalised for mental health disorders; aOR = 1.97 for having any parent hospitalised for DSH, all being significant at the level of p < .001. This study shows that timing is important for understanding intergenerational transmission of DSH risk. The pre-pregnancy period is as critical as period after childbirth for effective intervention targeting adult mental health disorders and DSH, highlighting the important role of adult mental health services in preventing DSH risk in future generations.

## Introduction

One in five children has a parent with a mental health disorder [1]. Children whose parents have mental health disorders are themselves at increased risk of developing a mental health disorder, including intentional self-injurious behaviours, with or without suicidal intent, known as deliberate self-harm (DSH) [2]. DSH is a major public health issue affecting 17% of people aged 15–24 years [3–5]. Risk factors for DSH in adolescents include maternal pregnancy and birth complications [6, 7], parental mental health disorders [8, 9], and social disadvantage [10–12]. However, limited research has investigated the impact of the timing of parental mental health disorders on adolescent DSH risk.

Previous research has shown that the earlier the child’s exposure to parental mental health disorders, the greater the impact on the child’s psychosocial development [13]. For example, maternal depression in infancy has a stronger influence on a child’s internalising disorders than maternal depression in toddlerhood [14]. Furthermore, children exposed to parental suicidal death in early childhood have an elevated risk of DSH-related hospital admissions, compared to parental suicidal death experienced in later childhood and adolescence [15].

Biological parenting may commence well *before pregnancy* [16]. This is because parental experiences prior to conception influence the development of the embryo and foetus, ultimately affecting the lifetime health of the child [17]. People with poorer mental health before pregnancy are more likely to have a higher number of health and social issues, which may elevate the risk of DSH behaviours among the adolescent children [18]. Women with mental health disorders before the conception of their child are more likely to have mental health distress in the pre and postnatal periods, compared to women who do not have pre-existing mental health conditions [19, 20]. *Perinatal period* is a critical window for children’s physiological and neurological development [21]. Perinatal exposure to environmental stress may cause epigenetic dysregulation, which may underlie the associations between adverse environmental exposures early in life and increased risk for children’s psychopathology [22]. Children exposed to heightened maternal mental health distress during the perinatal periods have elevated psychosocial problems during childhood [23, 24]. The mechanisms for maternal mental health problems during pre and postnatal period and increased psychosocial problems among children are complex and involve a range of biological and psychosocial risk factors that may be uniquely experienced early in life by the children, such as perinatal toxic stress, low attachment and poor parenting skills [25, 26].

*Childhood* is a period encompassing the critical development of the central nervous system in response to environmental exposures [26]. Childhood exposure to parental mental health disorders may result in increased emotional and behavioural dysregulation, which has been linked to heightened DSH risk in adolescence [27–29]. “Puberty is one of the central dramas of the human life course” [30], where substantive biological and psychosocial changes occur. *Adolescence* is a challenging time not only for adolescents themselves but also for parents, especially parents with mental health disorders [30–32]. The adolescent children of parents with mental health disorders are at especially high risk for DSH [10, 11].

Building on our previous study which only examined parental *lifetime* (not developmental period-specific) mental health disorders and/or DSH [7], we aimed to extend what is known about parent-child transmission of DSH risk by specifically investigating the effect of the *timing* of parental mental health and/or DSH-related hospital admissions on the child’s DSH risk in adolescence. Understanding the impact of the timing of parental mental health disorders is important in understanding the intergenerational transmission of mental health disorders, including DSH, and it can inform effective intervention and prevention strategies. To date, no research has investigated this issue. In this study, the child’s life course was partitioned into four stages: (1) pre-pregnancy, (2) pregnancy and infancy, (3) childhood and (4) adolescence, based on theoretical and empirical evidence described above.

## Materials and methods

As described above, this research advanced our previous study by examining the effect of the *timing* of parental mental health disorders and/or DSH-related hospital admissions on adolescent DSH risk [7]. In this study, we utilised the same administrative linked datasets, the same nested case-control sample generated from the same sampling strategy, and the same methods to identify exposures and outcomes.

### Record linkage

This study used the linked administrative data routinely collected by multiple government agencies in Western Australia (WA). The Data Linkage Branch (DLB) in WA managed the cleaning and extraction of information for linking records from different data collections that belong to the same person, by using unique identifiers (e.g., hospital unique medical record number or electoral number), record date, and demographic variables such as name, date of birth, residential address, and sex. Where the unique identifiers are not available across all data collections, the demographic variables are compared using a probabilistic matching method to calculate how likely records belong to the same person. Uncertain links are manually checked for validity by the DLB. A de-identified linkage key specific to each person is generated and stored in all data collections. The linkage accuracy is high: the proportion of invalid and missed links are estimated to be 0.11% [33]. The de-identified individual records across different data collections were merged using the linkage keys [34].

Four health related registers were linked for this study: the Hospital Morbidity Data System (HMDS) including all hospital inpatient admissions; the Mental Health Information System (MHIS) including mental health outpatients in public hospitals; the Emergency Department Data Collection (EDDC) including emergency department presentations in all hospitals under contract with the WA government; and the Death Registrations including all deaths registered in WA. Covariates were sourced from the Midwives Notification System (MNS) and the Birth Registrations that include all birth records in WA. All the administrative data collections date back to 1980 and earlier, except the EDDC which contains records since 2002.

### Study population

We established the source population using the Birth Registrations and the MNS, including all children born alive between 1981 and 2001 in WA identified as non-Aboriginal (244,104 males, 230,756 females). Given that DSH-related hospital admissions are a relatively rare event, with the overall prevalence being less than 2% [2, 11], a case-control sample was generated from the source population using nested case-control sampling strategy. Cases (n = 7,151) were composed of all the children who had a DSH-related hospital admission that occurred between the ages of 10 and 19 (inclusive) and no later than the year 2011. Each case was randomly matched with 20 controls by sex and year of birth. The matched controls (n = 143,020) were sampled from the source population, and they must not have had any DSH-related hospital admission or not have died by the date of the first DSH-related hospital admission of the matched case. Parental records were linked to the child’s information by the DLB through the Family Connection Systems.

### Adolescent first DSH-related hospital admission (outcome)

The outcome for this study was the child’s first DSH-related hospital admission that occurred between the ages of 10 and 19 during the study period (1981–2011). DSH-related hospital admissions were identified from the HMDS and the EDDC, and DSH-related deaths were identified from the Death Registrations. The identification of DSH-related hospital admissions was based on the International Statistical Classification of Diseases and Related Health Problems, 8^th^/9^th^ Revisions, Clinical Modification (ICD-8/9-CM: E950-E959), and the 10^th^ Revision, Australian Modification (ICD-10-AM: X60-X84). The convention in previous research was followed to include the “events with undetermined intent” (ICD-8/9-CM: E980-E989; ICD-10-AM: Y10-Y34) due to possible under-recording of DSH episodes in clinical settings (Hawton & Fortune, 2008). For records in the EDDC, the presenting symptom, the major diagnostic category, and the human intent of injury were used to identify any DSH-related emergency attendances.

The first DSH-related hospital admissions were mainly identified in the HMDS (52.3%), followed by the EDDC (45.9%). Of children with DSH-related hospital admissions, approximately 1% died during the study period (2.0% for males, 0.3% for females). Of the first DSH-related hospital admissions, 7.2% were coded as “event with undetermined intent” (8.7% for males, 6.4% for females).

### Exposure period

As described in the Study Population section above, we used an incidence density sampling strategy to create the nested case-control sample for the analysis. For each case and the 20 matched controls, we considered the time when the case child had his/her first DSH-related hospital admission as the end point of the observations for both the case and the 20 controls, and the case and the controls were matched by the date of birth. Therefore, there was an equal length of exposure interval allowing for the identification of parental admissions for the case and the 20 controls.

### Parental mental health and/or DSH-related hospital admissions (exposure)

Hospital admissions related to parental mental health disorders or DSH were identified from the HMDS, the MHIS, and the EDDC using the ICD codes (mental health disorders: ICD-8/9-CM: 290–319, ICD-10-AM: F01-F99; the same ICD codes as described above were used to identify parental DSH-related hospital admissions), and the presenting symptom and the major diagnostic category in the EDDC. This study focused on parental mental health and/or DSH-related hospital admissions that occurred prior to the end of exposure period. We combined maternal and paternal hospital admissions, this is because our preliminary analyses did not show maternal and paternal admissions had significantly different impacts on adolescent DSH risk.

**Fig 1:**
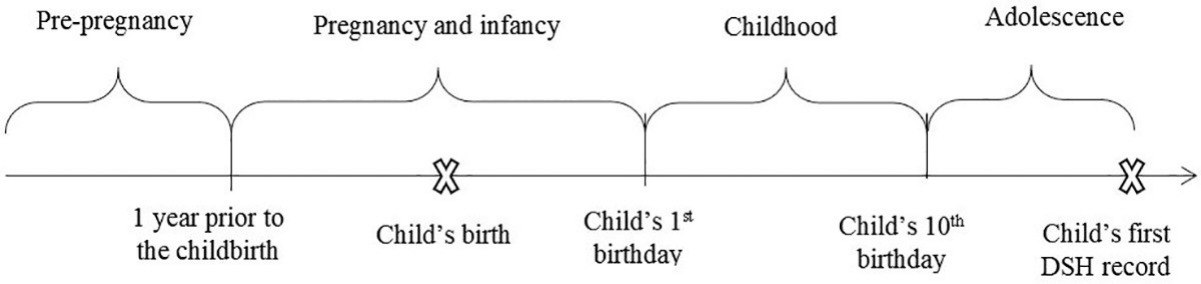
Timing of parental mental health and/or DSH-related hospital admissions.

The timing of children’s exposure to parental mental health and/or DSH-related hospital admissions was partitioned into four distinct time periods as shown in Fig 1.

The definition for each time period is:

1. Pre-pregnancy: the time period prior to one year before the child’s birth [35].
2. Pregnancy and infancy: the time period between one year before and one year after the child’s birth. We combined pregnancy and infancy to ensure an adequate sample size for robust statistical estimates (n≥16 for cases and n≥25for controls). Additionally, there has been a call for prenatal care to be provided in conjunction with postnatal care to parents with mental health disorders to ensure seamless perinatal care for the best possible start in life for their children [36, 37].
3. Childhood: the time period between the 1^st^ and the 10^th^ birthday. Childhood was not divided into early childhood (1–4 years) and late childhood (5–9 years), because our preliminary results did not show a significant difference between these two periods in the effects of parental mental health and/or DSH-related hospital admissions on adolescent DSH risk.
4. Adolescence: the time period between the 10^th^ and 20^th^ birthday [31].

### Covariates

The following groups of covariates associated with parental mental health disorders and adolescent DSH risk were identified [16, 17, 38] hence adjusted for this study. The first group was *child perinatal factors* including birth weight percentile by gestation, gestational age, and birth order [7]. The second group was *early maternal socio-demographic characteristics*, including maternal age and marital status at the time of the child’s birth, and the socioeconomic status (SES) of the neighbourhood where the mother resided at the time of the child’s birth. The age of father or the mother’s partner at childbirth was also available in the linked data, with 5% of the values being missing. We adjusted for paternal age in all the analyses, and the results largely remain unchanged. Regarding SES, only the variable reflecting the neighbourhood SES for mothers was available. However, 80% of the mothers in this study sample reported to be in a marital or a de facto relationship at the childbirth, thus this information would also largely reflect the neighbourhood SES for the fathers. The covariates in the first two groups were identified from the MNS and the Birth Registrations. The index of neighbourhood SES is developed by the Australian Bureau of Statistics [39], and it takes into account the income, educational attainment, and employment status of people residing in a census collection district containing approximately 250 dwellings.

Additionally, parental death due to any causes that occurred before the end of the exposure period was adjusted for as a covariate, and it was identified from the Death Registrations. Finally, children’s lifetime hospital admissions related to mental health disorders that occurred before the end of the exposure period were included as a covariate, using the same ICD codes and data source for the identification of parental mental health admissions described above.

### Data analysis and statistical methods

We first examined the frequency distribution for each covariate among cases and controls (Table 1). For each developmental period, we conducted conditional logistic regression analysis to estimate the change in the odds of adolescent DSH (i.e., odds ratio, or OR) associated with having a parent with a hospital admission related to a mental health disorder, DSH behaviours, and both, compared to having no parents with mental health or DSH-related hospital admissions. For each developmental period, we also compared the effect of having only one parent with a mental health hospital admission with the effect of having both parents with mental health hospital admissions on the odds of adolescent DSH. Because the number of children with both parents having a DSH-related hospital admission was very small, we only examined the effect of having any (i.e., at least one) parent with a DSH-related hospital admission on the odds of adolescent DSH (Table 2).

**Table 1:** Frequency distribution of covariates among cases and controls and the effects of covariates on the odds of adolescent DSH.

Covariates	Cases		Controls		aOR (95%CI) ^c^
	N.	%	N.	%	
**Total**	7151	100	143020	100	
**Sex** ^**a**^					
Male	2689	37.60	53780	37.60	n/a
Female	4462	62.40	89240	62.40	
**Age at the end of the study period (years)** ^**b**^					n/a
10–14	1592	22.26	31840	22.26	
15–19	5559	77.74	111180	77.74	
**Birthweight percentile by gestation (weeks)**					
<10% (small for gestational age)	827	11.56	14143	9.89	1.06 (0.95, 1.19)
10–25%	1163	16.26	22859	15.98	1.00 (0.91, 1.11)
25–50%	1820	25.45	36821	25.75	1.02 (0.94, 1.12)
50–75%	1700	23.77	35019	24.49	Reference
75–90%	973	13.61	20726	14.49	1.09 (0.98, 1.21)
> = 90% (large for gestational age)	666	9.31	13404	9.37	1.05 (0.93, 1.19)
Missing	2	0.03	48	0.03	n/a
**Gestational age (weeks)**					
20–36	426	5.96	7836	5.48	0.84 (0.74, 0.96) **
37–41	6488	90.73	131377	91.86	Reference
42–45	207	2.89	3294	2.30	1.21 (1.00, 1.47)
Missing	30	0.42	513	0.36	n/a
**Birth order**					
1	2750	38.46	56978	39.84	Reference
2	2344	32.78	48018	33.57	1.25 (1.15, 1.35) ***
3–4	1779	24.88	33840	23.67	1.43 (1.31, 1.56) ***
5+	274	3.83	4135	2.89	1.55 (1.29, 1.87) ***
Missing	4	0.06	49	0.03	n/a
**Maternal marital status at the child’s birth**					
Unmarried	1077	15.06	11264	7.88	1.03 (0.93, 1.14)
Divorced/Separated/Widowed	179	2.50	1410	0.99	1.50 (1.20, 1.88) ***
Married/De facto	5889	82.35	130276	91.09	Reference
Missing	6	0.08	70	0.05	n/a
**Maternal age at the child’s birth (years)**					
< 20 (teenage mother)	727	10.17	6818	4.77	1.61 (1.41, 1.84) ***
20–24	1940	27.13	30844	21.57	1.15 (1.06, 1.24) ***
25–34	3902	54.57	91631	64.07	Reference
35–39	499	6.98	11924	8.34	0.84 (0.75, 0.96) **
> = 40	82	1.15	1801	1.26	0.70 (0.53, 0.92) *
Missing	1	0.01	2	0.00	n/a
**Neighbourhood socioeconomic status**					
1 (<10%)–most disadvantaged	995	13.91	12474	8.72	1.14 (1.02, 1.27) *
2 (10–25%)	1145	16.01	19816	13.86	0.93 (0.84, 1.02)
3 (25–50%)	1699	23.76	33538	23.45	1.05 (0.96, 1.15)
4 (50–75%)	1405	19.65	30237	21.14	Reference
5 (75–90%)	673	9.41	17283	12.08	1.05 (0.94, 1.18)
6 (> = 90%)–least disadvantaged	445	6.22	11141	7.79	1.12 (0.98, 1.28)
Missing	789	11.03	18531	12.96	0.96 (0.86, 1.07)
**Parental all-cause death**					
No parents died	6839	95.64	139150	97.29	Reference
Only father died	195	2.73	2696	1.89	0.88 (0.73, 1.07)
Only mother died	111	1.55	1120	0.78	1.01 (0.78, 1.31)
Both parents died	6	0.08	54	0.04	0.65 (0.24, 1.79)
**Children’s mental health admissions**					
No	1886	26.37	134197	93.83	Reference
Yes	5265	73.63	8823	6.17	38.68 (36.09, 41.45) ***

**Table 2:** Distribution of having a parent with mental health and/or DSH-related admissions among cases and controls.

Parental admissions by cause and number of parents involved		Parental lifetime admissions		Timing of parental mental health and DSH-related hospital admissions							
				Pre-pregnancy		Pregnancy and infancy		Childhood		Adolescence	
Mental health admissions	DSH-related admissions	Case	Control	Case	Control	Case	Control	Case	Control	Case	Control
**No parent**	**No parent**	3491 (48.83)	104309 (72.96)	5340 (74.70)	126793 (88.68)	6480 (90.64)	136987 (95.81)	5063 (70.82)	124349 (86.97)	5364 (75.03)	128409 (89.81)
	**At least one parent**	97 (1.36)	990 (0.69)	143 (2.00)	1047 (0.73)	34 (0.48)	147 (0.10)	51 (0.71)	392 (0.27)	36 (0.50)	386 (0.27)
**Only one parent**	**No parent**	1848 (25.85)	26105 (18.26)	1081 (15.12)	11727 (8.20)	536 (7.50)	5121 (3.58)	1364 (19.08)	14168 (9.91)	1121 (15.68)	10509 (7.35)
	**At least one parent**	897 (12.55)	6384 (4.47)	397 (5.55)	2480 (1.73)	58 (0.81)	539 (0.38)	396 (5.54)	2426 (1.70)	465 (6.50)	2861 (2.00)
**Both parents**	**No parent**	370 (5.18)	3087 (2.16)	101 (1.41)	606 (0.42)	25 (0.35)	153 (0.11)	147 (2.06)	1049 (0.73)	85 (1.19)	549 (0.38)
	**At least one parent**	446 (6.24)	2097 (1.47)	87 (1.22)	319 (0.22)	16 (0.22)	25 (0.02)	128 (1.79)	588 (0.41)	78 (1.09)	258 (0.18)

The numbers in the parentheses refer to the percentages of children across all the categories in cases and controls respectively. For example, 48.83% (n = 3491) of the cases did not have any parents with lifetime mental health or DSH-related hospital admissions. Two cases and 48 controls did not have their parents’ records linked up, leaving 7149 cases and 142972 controls included in this analysis.

All the conditional regression analyses yielded ORs that were adjusted for the matching factors in the case-control design, including the child’s sex, year of birth, and age at the end of the exposure period. We conducted a series of multivariable regression analyses by progressively controlling for an increasing number of covariates. The modelling was completed in three steps. First (model 1), we adjusted for perinatal factors (birth weight by gestation, gestational age, birth order), early maternal socio-demographic factors (maternal age, maternal marital status, and maternal neighbourhood socioeconomic status at the time of the child’s birth), and parental all-cause deaths. Second (model 2), we *further* adjusted for parental mental health and/or DSH-related hospital admissions that occurred in other developmental periods. This was to control for the interdependence of parental mental health disorders and DSH behaviours in different developmental periods. Last (model 3), we *further* adjusted for children’s mental health hospital admissions in order to examine the extent to which the effects of parental admissions on the odds of adolescent DSH is accounted for by children’s mental health disorders, which has been shown to be the strongest covariate for DSH [2].

All covariates were treated as categorical factors following the conventional treatment of these factors in existing literature (see categorisations in Table 1). Two cases and 48 controls did not have their father’s records linked up, leaving 7,149 cases and 142,972 controls included in the analyses. P values less than 0.05 were considered statistically significant, using a two-tailed test. Confidence intervals at 95% level were calculated. All statistical analyses were conducted using SAS (EG) statistical software version 6.1 (SAS Institute Inc., Cary, NC, USA).

## Results

### Sample descriptions

Table 1 shows that compared to controls, cases (i.e., adolescents with a DSH-related hospital admission during the study period) were more likely to have a single, teen, or young mother, or a mother living in the most socioeconomically disadvantaged area at the time of the child's birth. Cases were much more likely to have been hospitalised for a mental health disorder than controls.

The prevalence of having at least one parent with a lifetime (i.e., prior to the end of the exposure period for the child) mental health hospital admission was 49.81% for cases, almost double that of controls (26.35%). The prevalence of having a parent with a lifetime DSH-related admission was 20.15% for cases, more than triple that of controls (6.63%). The prevalence of having a parent with mental health and/or DSH-related admissions among cases and controls for each developmental period is shown in Table 2. Generally, the prevalence for each period resembled that for lifetime parental mental health and/or DSH-related admissions.

### Effect of having a parent with a mental health hospital admission

In the sections below, we described the results in relation to having *only one* and *both* parents with mental health hospital admissions.

#### Having only one parent with a mental health hospital admission

Among children with a parent having a mental health hospital admission, the majority had only one parent with a mental health admission (cases: 83%, controls: 89% in Table 2). Table 3 shows that after controlling for all covariates in model 3, having *only one* parent with a lifetime mental health admission was associated with a 1.26-fold (95%CI: 1.16–1.36, p<0.0001) increase in the odds of having a DSH-related hospital admission during adolescence, compared to having no parents with either mental health or DSH-related hospital admissions.

**Table 3:** Effect of the timing of parental mental health hospital admissions on the odds of adolescent DSH.

Timing of parental mental health hospital admissions	Modelling ^b^	Number of parents having mental health admissions		Ratio of aOR ^c^
		One parent	Both parents	
**Lifetime**	**Models 1 & 2**	1.95 (1.83, 2.08) ***	3.05 (2.70, 3.45) ***	1.57 (1.38, 1.78) ***
	**Model 3**	1.26 (1.16, 1.36) ***	1.41 (1.21, 1.65) ***	1.13 (0.97, 1.33)
**Pre-pregnancy**	**Model 1**	2.02 (1.87, 2.17) ***	3.38 (2.69, 4.23) ***	1.67 (1.33, 2.11) ***
	**Model 2**	1.64 (1.52, 1.78) ***	2.34 (1.86, 2.96) ***	1.42 (1.12, 1.80) ***
	**Model 3**	1.25 (1.14, 1.37) ***	1.66 (1.23, 2.25) ***	1.35 (0.99, 1.84)
**Pregnancy and infancy**	**Model 1**	1.88 (1.70, 2.08) ***	2.99 (1.92, 4.68) ***	1.59 (1.01, 2.51) *
	**Model 2**	1.18 (1.06, 1.31) **	1.19 (0.75, 1.89)	1.00 (0.63, 1.60)
	**Model 3**	1.01 (0.89, 1.15)	0.73 (0.42, 1.27)	0.73 (0.42, 1.28)
**Childhood (1–9)**	**Model 1**	2.06 (1.92, 2.20) ***	2.60 (2.14, 3.15) ***	1.26 (1.04, 1.54) *
	**Model 2**	1.56 (1.45, 1.68) ***	1.44 (1.17, 1.77) ***	0.92 (0.75, 1.14)
	**Model 3**	1.15 (1.05, 1.26) **	0.88 (0.68, 1.12)	0.77 (0.60, 0.98) *
**Adolescence (10–19)** ^**d**^	**Model 1**	2.21 (2.05, 2.38) ***	2.77 (2.15, 3.58) ***	1.26 (0.97, 1.63)
	**Model 2**	1.69 (1.56, 1.83) ***	1.66 (1.27, 2.17) ***	0.99 (0.75, 1.30)
	**Model 3**	1.22 (1.11, 1.34) ***	1.05 (0.76, 1.46)	0.87 (0.62, 1.21)

^a^ Effects were measured in odds ratios (95% confidence interval in parentheses) derived from conditional logistic regression analysis, in reference to adolescents of unaffected parents during specific developmental periods (i.e., no mental health or DSH-related hospital admissions).

^b^ Model 1: Adjusting for perinatal factors (gestational age, birth weight percentile by gestation, birth order), early maternal socio-demographic factors (maternal marital status, maternal age, neighbourhood socioeconomic status at the time of the child’s birth), parental all-cause deaths; Model 2: Further adjusting for parental mental health and/or DSH-related admissions in other developmental periods, except for the effects of lifetime parental mental health admissions; Model 3: Further adjusting for children’s lifetime mental health admissions.

^c^ These results refer to the ratios of aORs associated with having both parents with mental health admissions to having only one parent with mental health admissions. For example, the first number 1.57 refers to the ratio of 3.05 to 1.95.

^d^ Parental mental health admissions during the child’s adolescence must occur prior to the end of the observation for that child.

* p < 0.05

** p<0.01

*** p<0.001.

For each developmental period, the effect of having *only one* parent with a mental health admission on the odds of adolescent DSH was significantly, yet largely attenuated, after controlling for parental mental health and/or DSH-related admissions that occurred in other developmental periods in model 2 (Table 3). After controlling for children’s mental health hospital admissions in model 3, having only one parent with a mental health hospital admission before pregnancy, during childhood, and during adolescence was associated with a 1.25-fold (95%CI: 1.14–1.37, p<0.001), a 1.15-fold (95%CI: 1.05–1.26, p<0.01), and a 1.22-fold (95%CI: 1.11–1.34, p<0.001) increase in the odds of adolescent DSH respectively, compared to having no parent with either mental health or DSH-related hospital admissions during that specific developmental period (Table 3).

#### Both parents with a mental health hospital admission

Table 3 shows that after controlling for all covariates in model 3, having *both* parents with a lifetime mental health hospital admission was associated with a 1.41-fold (95%CI: 1.21–1.65, p<0.001) increase in the odds of adolescent DSH, compared to having no parents with either mental health or DSH-related hospital admissions.

The effects of having both parents with a mental health hospital admission before pregnancy, during childhood, and during adolescence had a significant, yet largely attenuated, effect on the odds of adolescent DSH, after controlling for parental mental health and/or DSH-related hospital admissions in other developmental periods in model 2. However, after further controlling for children’s mental health hospital admissions in model 3, only having both parents with a mental health hospital admission *before pregnancy* was significantly associated with the odds of adolescent DSH (aOR = 1.66, 95%CI: 1.23–2.25, p<0.001) (Table 3). Compared with having only one parent with a mental health admission, having both parents with a mental health hospital admission did not have a significantly stronger effect on the odds of adolescent DSH, when controlling for all covariates in model 3 (ratios of aORs in Table 3).

### Effect of having a parent with a DSH-related hospital admission

Table 4 shows that after controlling for all covariates in model 3, having any parent with a lifetime DSH-related hospital admission was associated with a 1.63-fold (95%CI: 1.22–2.18, p<0.001) increase in the odds of adolescent DSH, compared to having no parents with either mental health or DSH-related hospital admissions.

**Table 4:** Effect of the timing of parental DSH-related hospital admissions on the odds of adolescent DSH.

Timing of parental DSH-related hospital admissions	Modelling ^b^	aOR (95%CI)
**Lifetime**	**Models 1 & 2**	2.46 (1.96, 3.09) ***
	**Model 3**	1.63 (1.22, 2.18) ***
**Pre-pregnancy**	**Model 1**	2.60 (2.15, 3.14) ***
	**Model 2**	2.15 (1.77, 2.61) ***
	**Model 3**	1.97 (1.53, 2.55) ***
**Pregnancy and infancy**	**Model 1**	3.01 (2.01, 4.52) ***
	**Model 2**	1.83 (1.21, 2.77) **
	**Model 3**	1.20 (0.72, 2.02)
**Childhood (1–9)**	**Model 1**	2.35 (1.72, 3.21) ***
	**Model 2**	1.82 (1.32, 2.50) ***
	**Model 3**	0.98 (0.65, 1.47)
**Adolescence (10–19)** ^**c**^	**Model 1**	1.62 (1.13, 2.34) **
	**Model 2**	1.19 (0.82, 1.74)
	**Model 3**	1.16 (0.73, 1.83)

^a^ Effects were measured in odds ratios (95% confidence interval in parentheses) derived from conditional logistic regression analysis, in reference to adolescents of unaffected parents during specific development periods (i.e., no mental health or DSH-related hospital admissions).

^b^ Model 1: Adjusting for perinatal factors (gestational age, birth weight percentile by gestation, birth order), early maternal socio-demographic factors (maternal marital status, maternal age, maternal neighbourhood socioeconomic status at the time of the child’s birth), parental all-cause deaths; Model 2: Further adjusting for parental mental health and/or DSH-related hospital admissions in other developmental periods, except for the effects of lifetime parental mental health admissions; Model 3: Further adjusting for children’s lifetime mental health admissions.

^c^ Parental DSH-related admissions during the children’s adolescence must occur prior to the end of follow-up.

* p < 0.05

** p<0.01

*** p<0.001.

Parental DSH-related hospital admissions that occurred before pregnancy, during pregnancy and infancy, and during childhood were associated with a 1.82- to 2.15-fold increase in the odds of adolescent DSH, after controlling for parental mental health and/or DSH-related hospital admissions that occurred in other periods in model 2. However, only parental DSH-related hospital admissions that occurred before pregnancy remained to have a significant and strong effect on the odds of adolescent DSH (aOR = 1.97, 95%CI: 1.53–2.55, p<0.001), after further controlling for children’s mental health hospital admissions in model 3 (Table 4).

### Effect of having parents with both mental health and DSH-related hospital admissions

It should be noted that mental health and DSH-related hospital admissions did not necessarily co-occur in one parent. It may be the case that mental health admissions occurred in one parent, and DSH-related admissions occurred in the other. After controlling for all covariates, having parents with both lifetime mental health and DSH-related hospital admissions was associated with a 1.83-fold (95%CI: 1.66–2.02, p<0.001) increase in the odds of adolescent DSH, compared to having no parents with either mental health or DSH-related admissions. This effect was 44% (95%CI: 30–59%, p<0.001) higher than the effect of having parents with a lifetime mental health admission only (without DSH).

**Fig 2:**
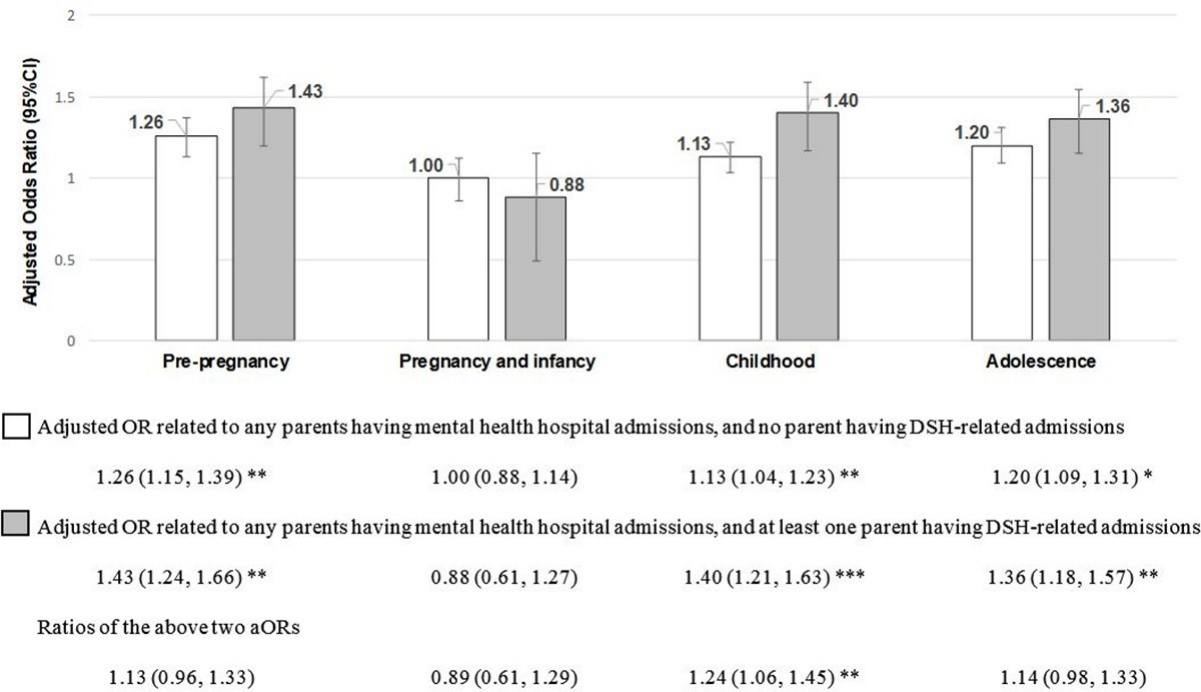
Effect of parental mental health and/or DSH-related hospital admissions on the odds of adolescent DSH.

Fig 2 shows that after controlling for all covariates, having parents with both mental health and DSH-related hospital admissions before pregnancy, during childhood, and during adolescence was associated with a 1.36 to 1.43-fold increase in the odds of adolescent DSH, compared to having no parents with either mental health or DSH-related hospital admissions in that specific period. Fig 2 also shows that having parents with both mental health and DSH-related hospital admissions before pregnancy, during childhood, and during adolescence was associated with increased odds of adolescent DSH, compared to having parents with a mental health admission only in that specific period. This increase was only significant for childhood (the ratio of aORs = 1.24, 95%CI: 1.06–1.45, p<0.01).

## Discussion

In line with previous research, this study showed that having a parent with a mental health hospital admission during childhood or adolescence increased adolescent DSH risk [10, 11]. Most importantly, this study has demonstrated that parental mental health disorders and/or DSH-related hospital admissions that occurred before pregnancy had a strong influence on children’s DSH risk in adolescence. This influence remained after controlling for important psychosocial covariates and parental mental health and/or DSH-related hospital admissions that occurred in pregnancy and infancy, childhood and adolescence. Moreover, this study has shown that parental mental health and/or DSH-related hospital admissions that occurred before pregnancy have a greater impact on adolescent DSH risk, compared to those that occurred during childhood and adolescence. We found that among children who had a parent with a mental health disorders and/or DSH-related hospital admission, nearly half of them had a parent experiencing such a hospital admission before the conception of the child. This is consistent with previous research showing that a preconception history of mental health problems was present in the majority of women who had perinatal depressive symptoms [20], suggesting that the effects of preconception mental health problems may be partly due to the chronicity of mental health disorders. Therefore, this study calls for adequate attention to preconception mental health in future research and interventions aimed at reducing the DSH risk among future generations.

People of reproductive age who have mental health disorders may receive inadequate preconception care or psychosocial support [40] which may increase the risk of pregnancy complications [41] and fetal growth restriction [42, 43], leading to increased risk of DSH in adolescence [6]. Further, psychosocial adversities associated with mental health disorders and DSH behaviours before pregnancy are likely to persist through pregnancy and into the postnatal period, affecting parenting capacities [16] and in turn the children’s mental health problems [44–46] and DSH in adolescence [47, 48].

This study has shown that having both parents with a mental health admission before pregnancy had a stronger effect on adolescent DSH risk than having only one parent with a mental health admission before pregnancy. Comparatively, the effect of having both parents with a mental health admission did not differ significantly from the effect of having only one parent on adolescent DSH risk in pregnancy and infancy, childhood and adolescence.

Another interesting finding in this study is that before pregnancy, during childhood, and during adolescence, parental DSH-related hospital admissions moderately increased adolescent DSH risk beyond the effect of parental mental health admissions, and this is in line with previous research [49]. Additionally, we found that before pregnancy, parental DSH-related hospital admissions had a stronger effect on adolescent DSH risk, compared to parental mental health admissions. These findings together may have suggested that before pregnancy, parental history of DSH may be a unique and stronger predictor for their children’s DSH risk in adolescence, compared to the history of mental health disorders.

Last, after controlling for parental mental health and/or DSH-related hospital admissions in childhood and adolescence and children’s lifetime mental health admissions, we did not find that parental hospital admissions occurring during pregnancy and infancy increased adolescent DSH risk. This may suggest that the effect of parental mental health problems during this period on adolescent DSH risk is partially accounted for by both children’s and their parents’ mental health disorders that occur during childhood and adolescence, which may have a more direct impact on adolescent DSH risk.

## Strengths and limitations

The greatest strength of this study is the use of linked administrative data, which has offered several advantages over traditional self-reported data. First, study samples are drawn from the whole population to minimise selection bias, which has been a major concern in survey studies on mental health [50]. Second, data are collected objectively using standardised clinical diagnoses (e.g., ICD codes) by clinical personnel. Third, long-term routine collections make the data on early exposure accessible, such as maternal socio-demographic factors during perinatal period, and parental mental health disorders and DSH behaviours in early developmental periods. Fourth, data linkage improves the identification of factors that may be perceived as stigmatising, such as mental health disorders and DSH behaviours, which are subject to under-reporting in survey data. Last, data linkage assembles life-course events in a chronological order more accurately than self-reported data, and this establishes more robust time sequences for making causal inferences.

The results of this study should be interpreted in light of several limitations. First, mental health disorders and DSH behaviours were only able to be identified if a person presented at a hospital or an emergency department. The information about mental health related consultations with a general practitioner, or a psychiatrist/psychologist who works in private practice, and prescriptions of medications for the treatment of mental health disorders are not available in the linked data. Therefore, the results in this study only reflect the impact of more severe parental mental health disorders and/or DSH behaviours on adolescent DSH risk [51]. Additionally, outpatient records in private hospitals are not available in the linked data. However, this may only have a small impact on the results, because private hospitals in Australia primarily provide inpatient care, which has been captured by the HMDC dataset used in this study [52].

Second, due to the small sample size, pregnancy and infancy were aggregated into one period in this study, in order to improve statistical power for robust estimates. However, it should be noted that these are two distinct periods, and there may be different mechanisms underpinning the influence of parental mental health problems that occur in different periods on the child’s mental health. Therefore, these two periods should be examined separately in future research.

Third, emigration records were not available in the linked data. An estimated rate of emigration from WA was under 2% in the general population [53]. We could not identify specific individuals who emigrated from WA during the study period, thus mental health or DSH-related admissions recorded outside WA could not be accessed.

Fourth, only parental mental health admissions that occurred after the year 1966 could be identified, which means that parental mental health and/or DSH-related admissions may be under-reported. This may be particularly an issue for parental admissions before pregnancy and may result in the underestimation of the effect of parental admissions before pregnancy on adolescent DSH risk.

Fifth, no information was available to indicate the presence of suicidal intent in the linked administrative data, therefore we could not identify whether suicidal intent was involved in a DSH admission. Last, the information about whether the children resided with their parents in each developmental period was generally not available in the administrative data used for this study. Therefore, we could not differentiate certain environmental influence (e.g., parent-child interactions, family functioning) from biological influence (e.g., genetic predisposition to mental disorders and/or DSH).

## Conclusion

This study showed the pervasive intergenerational effects of DSH risk over time. The intergenerational effects were evident before adults became parents, and persisted when their children reached adolescence. The findings call for mental health promotion and intervention policies and practices that extend across lifetimes. This extended vision of the life-course approach should target mental health before pregnancy, which is far earlier than the period considered in current intervention strategies and policies aimed at reducing adolescent DSH risk. Further, integrated child and family mental health services should begin in pregnancy and provide continuous support for children as they grow up. The capacity for service systems to operate intergenerationally depends on the extent to which services are integrated across different stages of the life course, and how well transitions between child, adolescent and adult mental health services are managed [54]. Rigorous investigation is needed to establish the mechanisms underlying the effect of the timing of parental mental health disorders and DSH behaviours on adolescent DSH risk.
